# Near-Peer Teaching to Integrate Anatomical Imaging into the Medical School Curriculum

**DOI:** 10.1007/s40670-026-02760-1

**Published:** 2026-06-03

**Authors:** Andrew E. Warfield, Curtis A. Plante, Ryan Walsh, Abigail Hielscher

**Affiliations:** 1 Larner College of Medicine, University of Vermont, Burlington, VT USA; 2 Department of Radiology, University of Vermont, Burlington, VT USA; 3 Department of Neurological Sciences, University of Vermont, Given D-403 89 Beaumont Ave, Burlington, VT 05405 USA

**Keywords:** Near-peer, Anatomy education, Radiology education, Medical student, Pre-clinical, Imaging

## Abstract

**Purpose:**

Additional instruction in imaging can solidify medical students’ anatomical knowledge, which in turn, supports better clinical reasoning skills. Imaging education is often constrained due to insufficient time in the curriculum as well as instructor availability. Near-peer teaching can address some of these constraints.

**Methods:**

Imaging at our school is integrated across seven anatomy blocks in the first-year curriculum. One-hour optional review sessions, led by second-year medical students, were incorporated into each block with a focus on high yield exam concepts. Attendance was collected at each session. Pre- and post-session quizzes, post-session surveys, and an end of course questionnaire measured student knowledge and perceptions. Quiz performance was compared before and after each session. Survey responses were recorded on a Likert scale and quantitatively analyzed. Open ended questions were analyzed using thematic analysis.

**Results:**

An average of 50.7 ± 6.9 students attended each session and students performed better on the post-session quizzes for 6/7 sessions. Students felt more knowledgeable about anatomy and imaging topics and were more prepared to answer test questions following these sessions. Longer-term knowledge retention, assessed at the end of the course using multiple choice questions from each pre/post-session quiz, was also evident. Students felt satisfied with near-peer facilitators and with session content and delivery. Comments focused on desiring more time to answer practice questions, acquiring learning tips from facilitators, and preferring longer sessions.

**Conclusions:**

This study demonstrates that near-peer-led imaging sessions are feasible, engaging, and have positive effects on student knowledge and test preparedness.

**Supplementary Information:**

The online version contains supplementary material available at https://doi.org/10.1007/s40670-026-02760-1.

## Introduction

Current medical school education in imaging is lacking [1–6]. A 2018 national survey of 100 randomly selected program directors spanning multiple specialties found substantial trainee knowledge gaps for both image interpretation and utilization skills [2]. This may lead to delays, increased costs and unnecessary testing for patients [2]. Trainees also feel these inadequacies in their imaging education. National surveys in both the United States and the United Kingdom indicate that fourth-year medical students and first-year residents along with clinical educators believe there is too little radiology instruction in medical school [3–6]. Similarly, a national survey of first through fourth year medical students in United States based medical schools found that most students surveyed perceived their imaging training was lacking [6]. Together, these studies indicate that both students and clinician educators believe that medical students aren’t receiving sufficient imaging training to meet clinical needs.

Two main barriers exist to introducing more imaging education into the preclinical medical school curriculum. First, the curriculum is already saturated with limited instructional time and second, the availability of radiologists is limited [7]. Additional education requirements would also strip time from radiologists’ clinical practice. Currently, most imaging education occurs in fourth-year elective rotations which bypass these barriers [7, 8]. However, these rotations are mainly utilized by students who plan to match into radiology residency, curtailing their benefit to trainees in other areas and limiting exposure to the field for students who may otherwise be interested.

One method to limit oversaturating the curriculum is to integrate preclinical imaging education with gross anatomy, as imaging acumen relies on sound anatomical knowledge. Several previous studies have shown the advantages of blending anatomy with imaging education. These include better student engagement, stronger radiologic skills, more effective learning of anatomy, and longer knowledge retention [9–16]. In support of integrating anatomy with the imaging curriculum, several groups have found that students feel more engaged and confident when anatomy concepts are presented with both cadaveric donors and imaging [13–16].

Near-peer teaching, or teaching directed by students who are more advanced in their program of study, offers a way to integrate additional imaging education. Many studies have shown that both student-learners and student-teachers benefit from peer teaching [17–22]. Student-learners benefit from a uniquely informed teaching style while student-teachers build on their knowledge, grow as leaders, and practice teaching skills [22]. Two previous studies on near-peer teaching and imaging in preclinical medical school education have shown positive opinions from students and near-peer facilitators with most students finding these sessions very helpful [21, 22]. However, neither of these studies attempted to assess student learning or longer-term knowledge retention. The current study presents an ongoing near-peer teaching program aiming to build foundational imaging skills early in the preclinical curriculum at our institution. As a theoretical framework for our study, we applied both cognitive congruence, which relies on near-peers having a better understanding of learner’s knowledge and their needs based on proximity in training, and social congruence, which relies on learners perceiving their near-peers to be more approachable and relatable than faculty for addressing their learning gaps [23–26], to guide our intervention. In this program, two second-year medical students (M2s), authors on the manuscript, worked with a faculty anatomist and faculty radiologist to design and lead 7 one-hour long teaching sessions for first-year medical students (M1). These near-peer led sessions took place during the Foundations of Clinical Sciences (FoCS) course, which introduces students to several disciplines within the basic sciences including anatomy and imaging. The goal of this study was not only to promote M1 engagement in imaging and address any student-perceived learning gaps, but to assess the impact of the near-peer facilitated sessions on both short-term and longer-term knowledge retention. In addition to student knowledge, exam preparedness, satisfaction, and feedback were assessed throughout and at the end of the near-peer teaching program.

## Materials and Methods

### Ethics Statement

The Institutional Review Board, Ethics Committee on Human Research at our institution reviewed this study and determined it to be exempt from full review. M1 students were informed of the nature of the study via communication within their course calendar. Here, students viewed study information and session materials. Responses to questionnaires were voluntary and participation didn’t affect a student’s academic standing.

### Anatomy Course Details

Anatomy is taught to M1 students as part of the larger FoCS curriculum along with several other foundational disciplines. This takes place over the course of four months in the first semester of the M1 curriculum. FoCS is delivered in 7 blocks: Back and Shoulder (block 1), Upper Limb (block 2), Thorax (block 3), Abdomen (block 4), Head and Neck (block 5), Pelvis and Reproductive System (block 6) and Lower Limb (block 7). Within each block, students learn the relevant anatomy, embryology, histology and imaging. For further description of the anatomy curriculum at LCOM, please refer to Brown et al. [27].

### Imaging Curriculum Details

The imaging curriculum consists of seven interactive pre-work modules and seven ninety-minute active learning sessions. Pre-work imaging modules include didactic and interactive components that students can work through at their own pace. The imaging pre-work modules were created in PowerPoint by a team of radiologists including one attending (an author of this study) and several residents. The PowerPoint was transferred to Articulate 360 (Waltham, Massachusetts) by a member of our institution’s active learning team. Articulate 360 was used to add interactive elements including embedded instructional videos and quizzes in which students drag/drop anatomical structures to their correct location on radiographs, CTs, MRIs and angiograms. Active learning sessions employed the use of Pacsbin (Orion Medical Technologies, Baltimore, MD), a web-based DiCOM viewer with free and low-cost subscription plans based on case volume need. Students, working in small groups of up to 6 members, logged into Pacsbin using a provided login specific to their table number, and labeled anatomical structures from de-identified patient images uploaded to the system. In general, imaging instruction in both the pre-work and active learning sessions relied on identification of anatomical structures using radiographs, CTs, MRIs and angiograms. Since our curriculum is focused on body regions, we aligned the imaging component to complement the regional anatomy. For example, when students were learning about the deltoscapular region in the anatomy lab, they were also identifying this anatomy in their imaging session. Anatomical labeling was tied to certain imaging modalities to highlight strengths and weaknesses of these as related to differences in tissue composition. Regarding musculoskeletal anatomy, radiographs and CTs were used to illustrate osseous features, CT and MRI for the identification of soft tissue structures (muscles, tendons and ligaments) and angiograms for vascular anatomy. Although the focus of the active learning sessions was on normal anatomy, a few basic pathology cases were utilized to bring clinical context into their learning. For additional details about our imaging curriculum, please refer to Afshari et al. [21].

### Educational and Learning Objectives

The following highlights the overall educational objectives of the study along with some learning objectives from active learning sessions that were applied to near-peer teaching sessions. For further details on learning objectives used in both imaging and anatomy sessions, please see Supplementary Fig. 1.

Educational Objectives

1. Review and refine student’s anatomical and imaging knowledge. For M1s this was accomplished through engagement in sessions, allowing concept review and closure of knowledge gaps. For M2s, the objective was accomplished through review of faculty-designed materials as well as conversations with faculty allowing M2s to review their anatomical and imaging knowledge prior to instruction.
2. Provide additional exposure to imaging through incorporation of multiple imaging modalities. This gave students an additional opportunity to learn more about imaging and its applications.
3. Incorporate the knowledge and experience of near-peers to design and deliver session materials. This objective was accomplished with a dedicated and enthusiastic team of M2 students who worked carefully with faculty to design and deliver effective review sessions.

Learning Objectives

1. Identify the different anatomical planes
2. Recognize advantages of different imaging modalities
3. Identify structures of each anatomical region (e.g. back, deltoscapular region, upper limb, etc.) on radiographs, CTs, MRIs and angiograms

### Attendance

Sign-in sheets were distributed at the start and end of each session. A sign-in sheet was not available for block 1 and the number of post-session quiz responses was used to estimate attendance.

### Development of Session Materials

Session materials and quizzes were designed by two M2 students and reviewed by a faculty anatomist and faculty radiologist for accuracy and completeness. In particular, the anatomy faculty member reviewed anatomical content as it related to the accuracy and diversity of region-specific topics covered in the pre/post-session quizzes and additionally evaluated the accuracy of information related to anatomical details in the PowerPoint materials. In some instances, students were asked to identify a radiologic structure and then were asked follow-up anatomical questions (e.g. identify the bony structure and tell us what nerve travels near the structure). These types of questions were also reviewed by the anatomy faculty member. The radiologist faculty reviewed images and their associated labels for accuracy. Both faculty members provided feedback to M2 students regarding how the content in their session materials correlated with what was being taught to the M1s. M2 students used curricular elements as a general guide and added learning tips and tools where appropriate. All M1 students, regardless of attendance, were able to access session materials on their curricular calendar. M2s did not have any imaging specific training prior to running these sessions but chose to conduct these sessions based on interest in the field and a desire to teach.

Session materials were created with the goal of integrating anatomical knowledge and imaging knowledge together and as such the bulk of the session slides consisted of radiological images with anatomical structures marked for students to identify. This was paired with additional questions allowing students to test their knowledge on the function, innervation or pathologies related to these anatomical structures. Supplementary Fig. 1 illustrates the timing of faculty-led anatomy, faculty-led imaging, near-peer teaching sessions and exams and further shows how the learning objectives of the anatomy and imaging sessions were covered in near-peer led sessions. It should be noted that near-peer teaching sessions either happened shortly after faculty-led imaging sessions or on the day of imaging sessions. Near-peer led sessions which coincided with the same day as faculty-led imaging sessions were a result of room availability constraints and timing of other extra-curricular noon time activities on preferred days. M2 students developed the content for each of the 7 anatomical imaging review sessions and co-led each of the 7 review sessions. A near-peer-led session activity from the PowerPoint on the Thorax anatomy block is available in Supplementary Fig. 2.

### Sessions

Each of the 7 hour-long review sessions were facilitated by M2 students. These near-peer-led review sessions were held in medical education classrooms equipped with several monitors. Each session focused on one anatomical region (e.g. upper limb). A pre-session quiz, consisting of 7–10 multiple choice questions covering content relevant to the block, was administered prior to the review. The same quiz was administered at the end of the review (post-session) to gauge students’ short-term knowledge retention. Students were given up to 10 min each to complete the pre- and post-session quizzes with 40–45 min of review between. Quizzes were administered using Qualtrics XM version 2020 (Qualtrics, Provo, UT), a web-based survey tool available free of cost to faculty and students at our institution. Reviews were delivered using PowerPoint, where students worked in small groups to label radiographs, CT scans or MRI images relevant to the block. All radiographic images were courtesy of IMAIOS © "Micheau A, Hoa D, e-Anatomy, www.imaios.com, https://doi.org/10.37019/e-anatomy”. Interwoven in the imaging were practice questions that asked students to apply their anatomical knowledge toward clinical scenarios. Correct answers were then revealed, and students could ask follow-up questions. After completing the post-session quiz, students were invited to take a brief post-session survey, delivered via Qualtrics XM. All results were obtained anonymously. Supplementary Fig. 3 shows the quiz questions used for each session along with the post-session survey.

### End of Course Questionnaire

One month following the conclusion of FoCS, M1 students received an email invitation to complete the end of course questionnaire (ECQ), administered using Qualtrics XM. The questionnaire assessed knowledge retention and students’ perceptions on the effectiveness of sessions. The first component of the questionnaire included 14 multiple choice questions such that 2 questions from each of the 7 pre- and post-session quizzes were included (Supplementary Fig. 4). Questions were selected by M2 facilitators based on the following metrics: representation of anatomy and imaging learning objectives, similarity to USMLE STEP 1 questions, and likelihood of recurrence of content on USMLE STEP 1 exam. The second part of the questionnaire consisted of 13 items of which 12 were presented on a 5-point Likert scale (Supplementary Fig. 4). The last question was open-ended, allowing for student narrative feedback (Supplementary Fig. 4).

### Data Collection and Graphs

Data on pre- and post-session quizzes was collected over the four-month time span that sessions took place. Data from pre- and post-session quizzes were unmatched. We didn’t collect data in a manner that was identifiable in nature or that used codes to link pre- and post-session quiz sets. The intervention was open to all interested medical students and as such, did not exclude students. In this manner, we didn’t have a control group or a group who didn’t receive the intervention for this study. The ECQ data was collected over a two-week time span at the start of the second semester (about a month after first semester break). All information from questionnaires and quizzes was obtained using Qualtrics XM. Anonymous participant responses were saved, analyzed and converted to graph format in a Microsoft Excel spreadsheet (Microsoft, Redmond, WA).

### Positionality Statement

The M2 students who prepared and facilitated review sessions also analyzed data relevant to the present study. Both students have an interest in imaging and were thus motivated to prepare and facilitate sessions with the goals of addressing M1 learner knowledge gaps as well as increasing their knowledge in a field in which they intend to pursue as a future career. The concern for bias was that more attention would be paid to positive comments than negative ones. For this reason, the decision was made to cluster student narrative feedback based on content areas mentioned in the narrative feedback (i.e. design of class materials, pre/post session quizzes)**.** As an additional measure to address bias, an outside reviewer skilled in survey design methods reviewed our surveys prior to IRB review and administration to M1 learners. This individual provided feedback on how our surveys could be revised for clarity and to reduce any potential bias.

### Thematic Analysis

A thematic analysis was conducted on open-ended questions from post-session surveys and the ECQ requesting feedback on ways to make the near-peer-led review sessions more successful in the future. A reflexive thematic approach was used to identify themes associated with narratives [28].

### Statistical Analysis

**Fig. 1:**
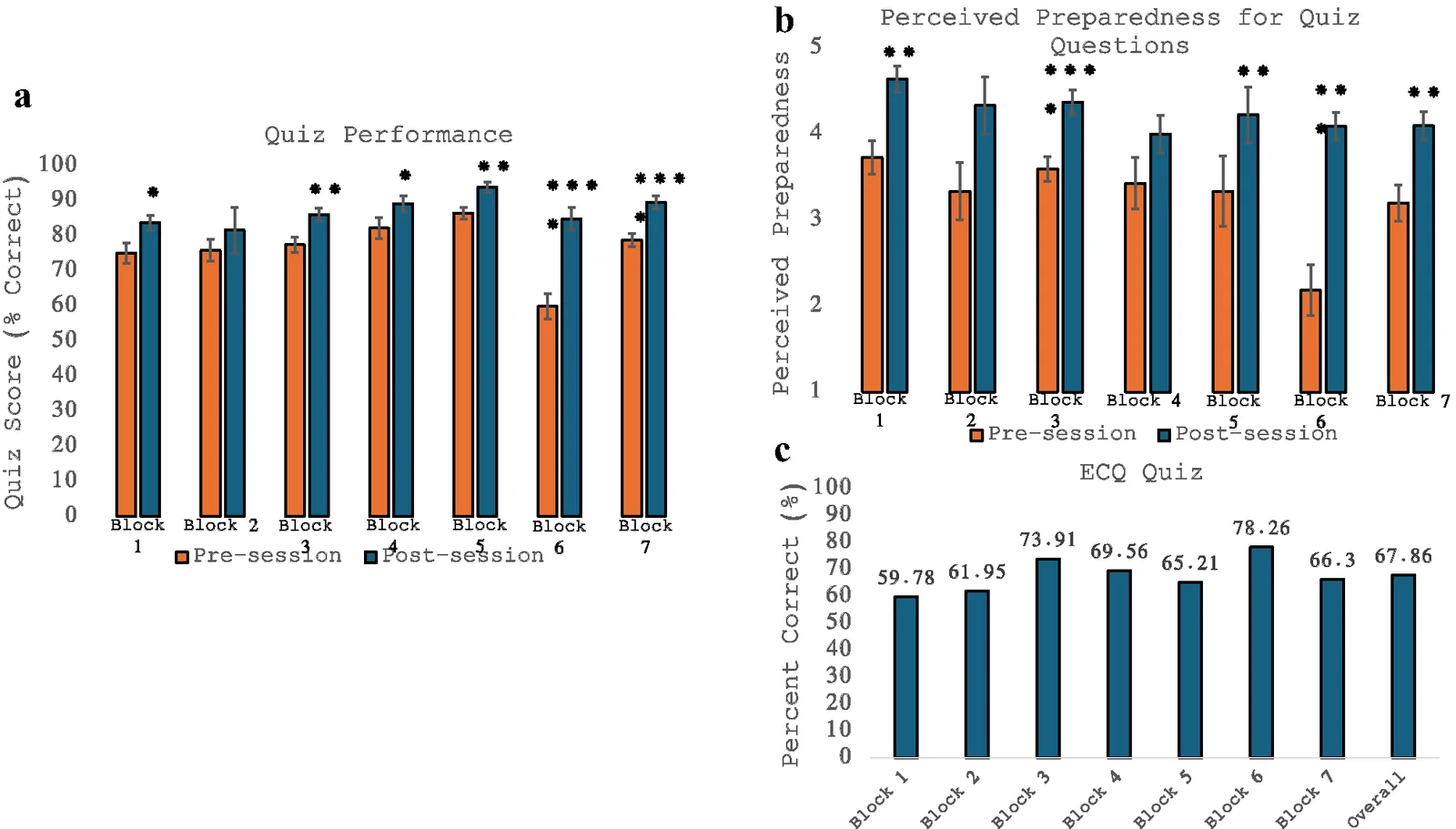
Performance Data on Assessments.** a** Student performance on the post-session quiz was significantly higher than on the pre-session quiz (orange) in six of seven blocks. Block 1 (back/deltoscapular region): *p = 0.0117 (*n* = 25 pre-session, 41 post-session). Block 2 (upper limb): ^ns^*p* = 0.0674 (*n* = 28 pre-session, 15 post-session). Block 3 (thorax): **p = 0.0027 (*n* = 45 pre-session, 56 post-session). Block 4 (abdomen): *p = 0.0436 (*n* = 38 pre-session, 44 post-session). Block 5 (head/neck): **p = 0.0023 (*n* = 51 pre-session, 31 post-session). Block 6 (pelvis): ****p < 0.0001 (*n* = 29 pre-session, 44 post-session). Block 7 (lower limb): ****p < 0.0001 (*n* = 36 pre-session, 30 post-session). **b** Student preparedness for quiz questions was significantly better post-session than it was pre-session in five of seven sessions. Here, 1 indicates “not ready at all”, 2 indicates “getting there”, 3 indicates “neutral”, 4 indicates “mostly ready” and 5 indicates “completely ready”. Block 1: **p = 0.0039 (*n* = 11). Block 2: ^ns^*p* = 0.2254 (*n* = 3). Block 3: ****p < 0.0001 (*n* = 27). Block 4: ^ns^*p* = 0.2500 (*n* = 7). Block 5: ****p < 0.0001 (*n* = 9). Block 6: ***p = 0.0010 (*n* = 11). Block 7: **p = 0.0078 (*n* = 10). **c** Student performance on the quiz questions as part of the ECQ (*n* = 46)

Statistical analyses for continuous variables were performed by exporting data from Excel to Prism 9 (GraphPad; San Diego, CA). Normality was assessed using a Shapiro–Wilk test. Normally distributed data were analyzed using unpaired two-tailed T-tests where appropriate. For non-normally distributed data, a Mann–Whitney U test was used for unpaired data, and a Wilcoxon signed-rank test was used for paired data (Fig. 1b only). When ordinal data from Likert scale (1–5) responses were compared statistically, they were assumed to have equal distance between options and analyzed accordingly (Wilcoxon Matched signed rank test used in Fig. 1b). Significance level was set at α = 0.05 for all analyses. Summary statistics were analyzed using frequency counts. If a comment included more than one theme it was counted towards two or more independent themes. A reflexive thematic approach was used to identify themes associated with narratives from question 27 in the ECQ and questions 6 and 7 from the post-session survey.

## Results

### Participants and Student Responses

Review sessions were open to all M1 students enrolled in FoCS. Attendance averaged 50.7 ± 6.9 students ranging from 41 to 59 per session. This gave an attendance rate ranging between 33.1% and 47.6% of the 124 M1 students.

There were 36.0 ± 9.5 pre-session quiz responses, yielding a response rate of 72.3 ± 14.2%, and 34.4 ± 13.6 post-session quiz responses, yielding a response rate of 64.4 ± 27.9%. Additionally, 11.14 ± 7.54 students responded to the post-session surveys, yielding a response rate of 21.2 ± 14.2%. For the ECQ, there were a total of 55 responses for a response rate of 44.4% (55/124).

### Student Knowledge

Students performed statistically better on post-session quizzes (87.02% ± 4.16 correct overall) than they did on pre-session quizzes (76.57% ± 8.34 correct overall) for each anatomical region except for block 2 (upper limb; Fig. 1a). Notably, block 2 had the lowest number of post-session quiz responses. Students who responded to post-session surveys indicated feeling more prepared for post-session quiz questions (Fig. 1b). To evaluate longer-term knowledge retention, we administered an ECQ one month after the conclusion of FoCS. The ECQ included 14 quiz questions used throughout the semester on pre/post session quizzes. The average score was 67.9 ± 15.3% with the lowest average block score coming from block 1-back and deltoscapular region (59.8%) and the highest from block 6-pelvis/perineum (78.3%, Fig. 1c). We did not find a significant correlation between students who attended more sessions and received higher scores on the quiz questions in the ECQ.

**Fig. 2:**
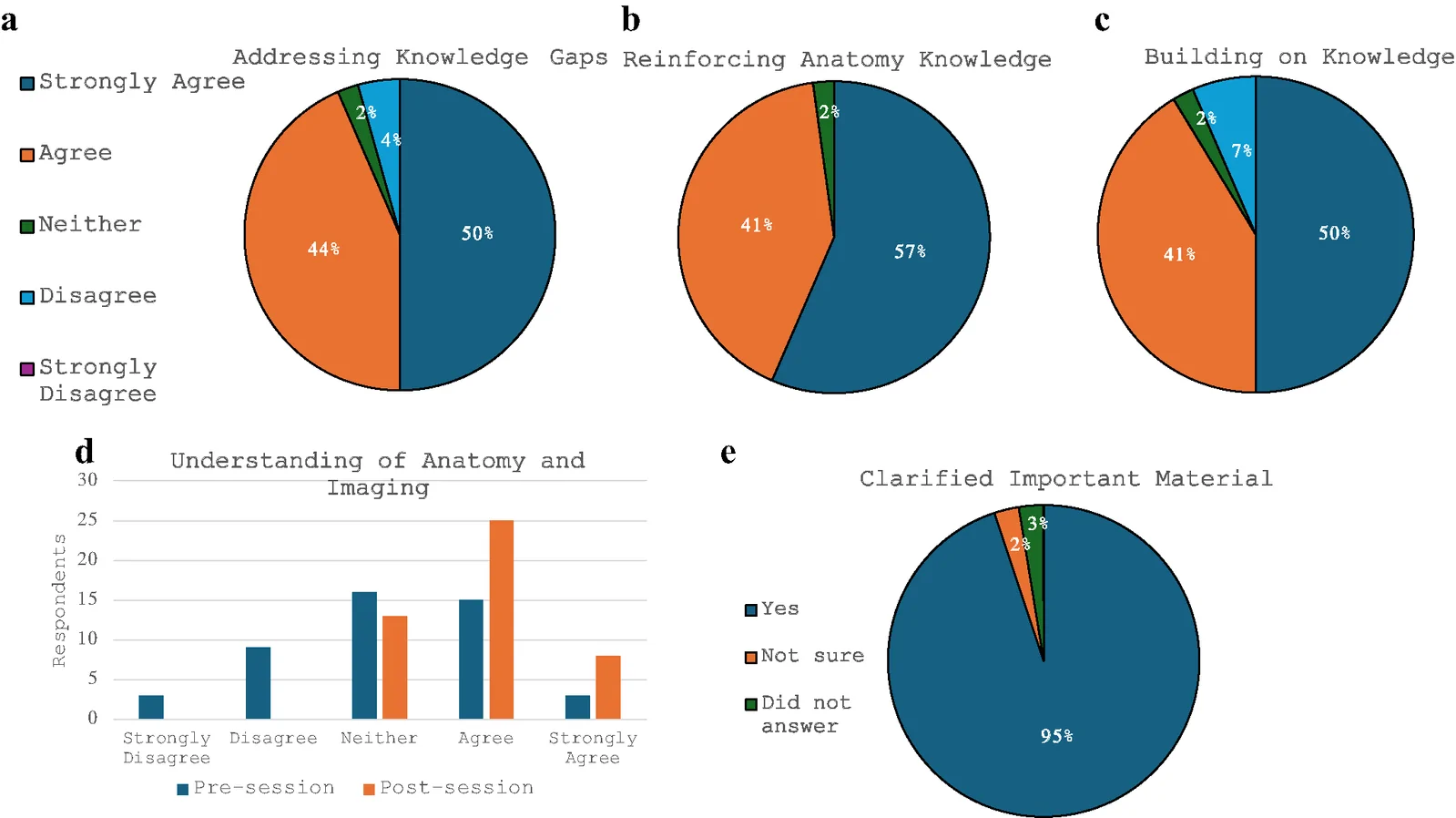
Students’ perceptions of their knowledge.** a**, **b**, **c**. Over 90% of students either strongly agreed or agreed that sessions addressed knowledge gaps, reinforced anatomy knowledge, and built on their anatomy knowledge. **d** Most students strongly agreed or agreed that they had a solid understanding of anatomy and imaging post-session more often than they did pre-session. **e.** Within the post-session surveys, students overwhelmingly reported that sessions clarified important material

In addition to performance metrics, we asked whether sessions addressed knowledge gaps, reinforced knowledge, and built upon prior knowledge (Fig. 2a-c). Students overwhelmingly agreed that sessions accomplished these goals with 94%, 98% and 91% either strongly agreeing or agreeing with each goal respectively. When asked about understanding anatomy and imaging topics, ECQ results showed that 71.7% (33/46) of students either strongly agreed or agreed that they had a solid grasp on the concepts following the sessions they attended as opposed to 39.1% (18/46) before sessions (Fig. 2d). Within post-session surveys we asked whether sessions clarified anatomical concepts and found that 95% (range: 92.6%−100%) of students responded “yes.” (Fig. 2e). Together, these data indicate that near-peer sessions led to significant short-term knowledge gain and that students on average retained much of this knowledge one month upon completion of the course. The data also indicates that the majority of students valued the sessions for reinforcing their anatomical and imaging knowledge and addressing knowledge gaps.

### Exam Preparedness

**Fig. 3:**
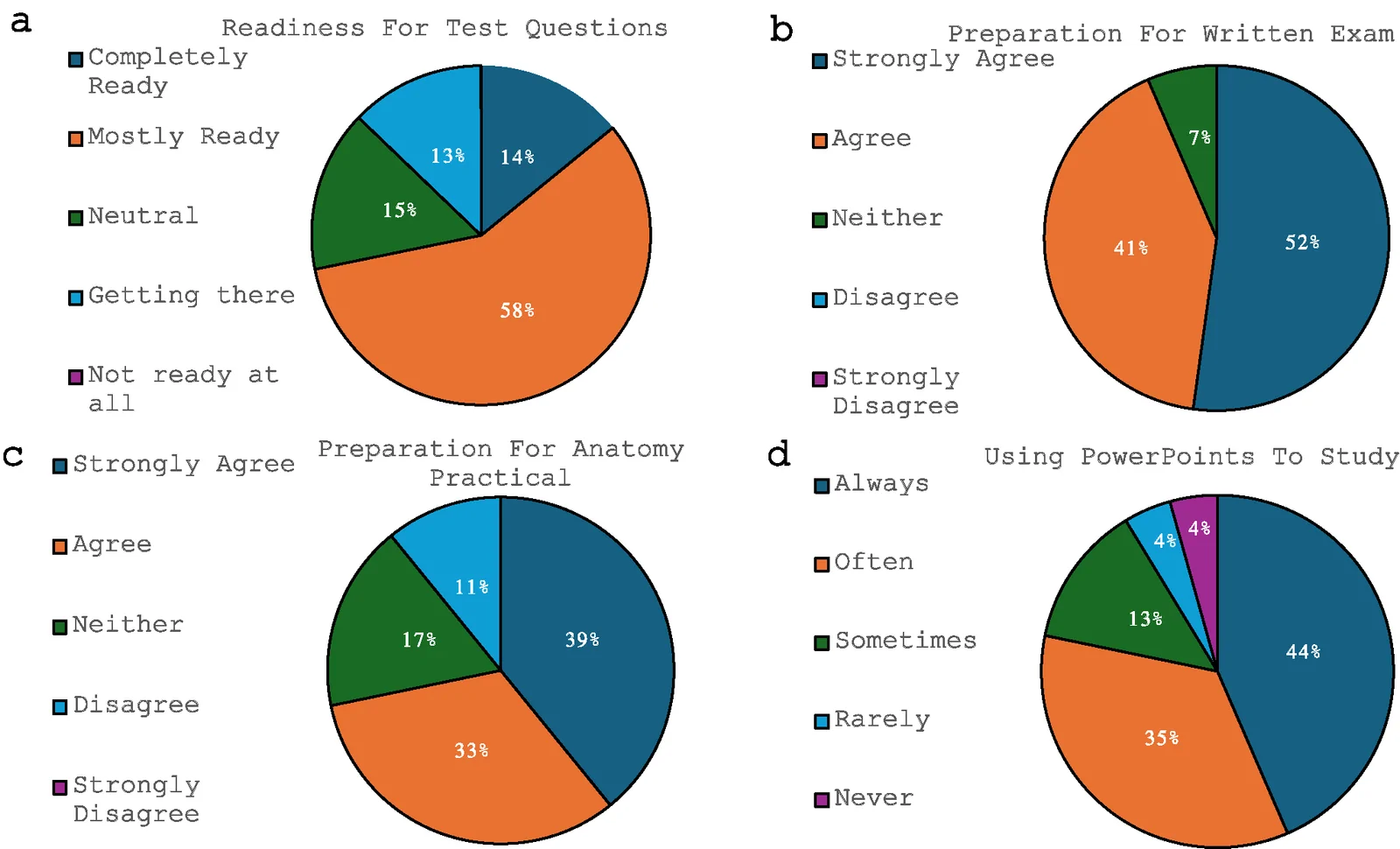
Students’ perceptions on exam readiness. **a** Students’ perceptions on exam readiness. Post-session survey results revealed that an average of 14% of students indicated being completely ready while 58% indicated being mostly ready for their block exams. **b** and **c** ECQ data demonstrated that most students believed the sessions helped them to prepare for written exams and to a lesser extent the anatomy laboratory practicals. **d** When asked how often session PowerPoints were used to study, the majority responded either “always” or “often”

We next assessed the impact these sessions had on students’ perceptions of their exam preparedness. According to post-session survey results, 14% (range: 0%−33.3%) of students indicated they were completely ready for exam questions testing anatomical and imaging concepts after participating in the sessions while 58% (range: 33.3%−70.4%) indicated they were mostly ready (Fig. 3a). As part of the ECQ, we asked students whether sessions helped them prepare for written exams (Fig. 3b) and anatomy laboratory practicals (Fig. 3c) and whether they used session PowerPoints to study for exams (Fig. 3d). Overall, 93% of students strongly agreed or agreed that sessions helped prepare them for written exams. Students felt the sessions were somewhat less helpful in preparing them for anatomy laboratory practicals with 72% strongly agreeing or agreeing. Finally, most students reported using session PowerPoints to study for exams with 44% indicating “always”, 35% indicating “often”, 13% indicated “sometimes”. This data indicates that students perceived these sessions to be beneficial toward exam preparation and further used these resources when preparing for exams.

### Student Satisfaction

**Fig. 4:**
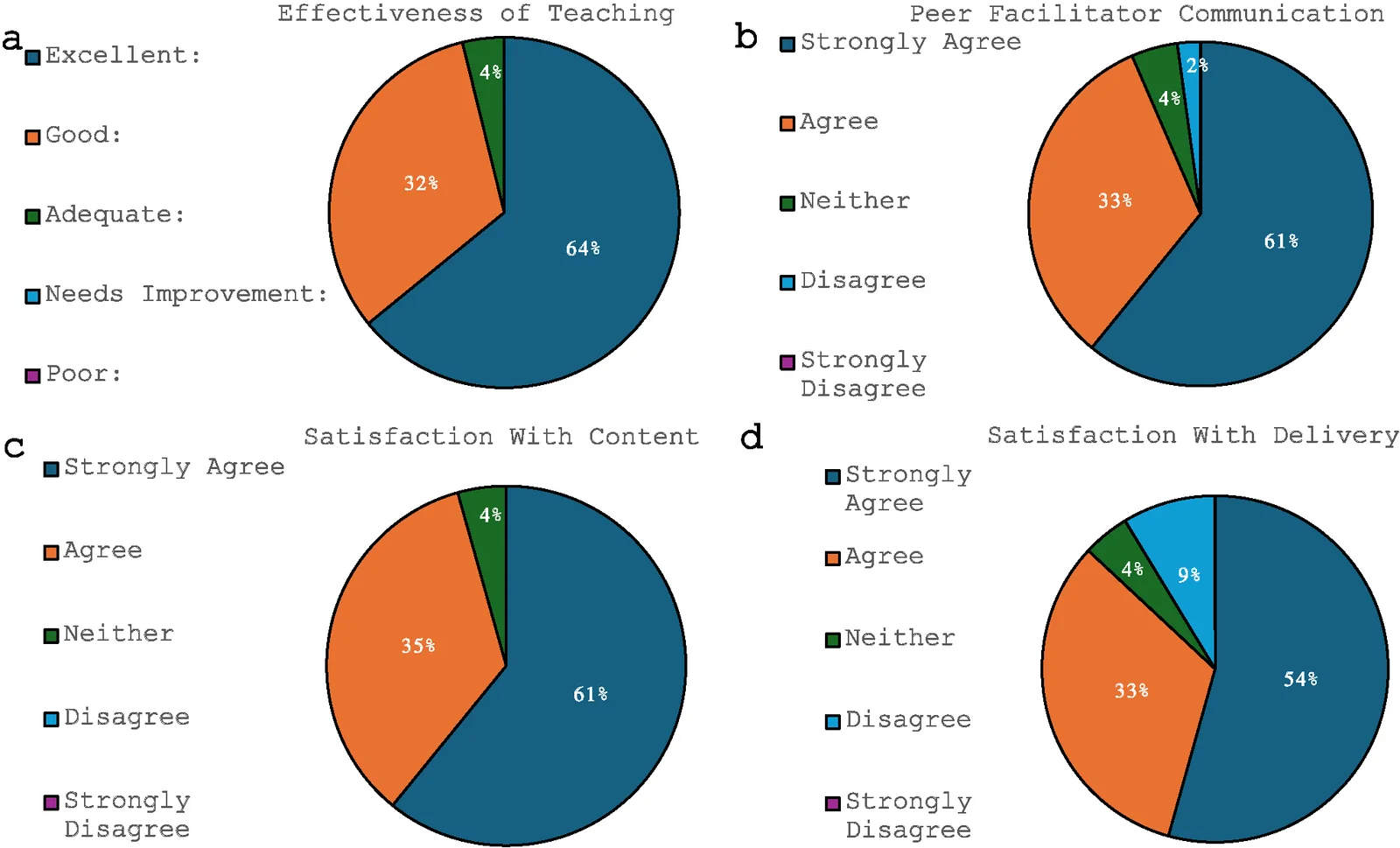
Student satisfaction. **a** Post-session survey results demonstrated that more than 90% of students felt the near-peer teaching was either excellent or good. **b** ECQ results demonstrated that 61% and 33% of students strongly agreed or agreed, respectively, that they were satisfied with near-peer communication. **c** and **d** ECQ results showed that 61% and 54% of students strongly agreed and 35% and 33% agreed that they were satisfied with the content and the delivery of the content, respectively

Students responded positively to near-peer teaching in the post-session surveys with 64% (range: 33% to 91%) indicating that the teaching was excellent and 32% indicating good (range: 10% to 67%; Fig. 4a). Peer-facilitator communication was also rated highly in the ECQ. Here, 61% of students strongly agreed and 33% agreed that communication was effective (Fig. 4b). Satisfaction with session content and delivery was high as well with 61% and 54% strongly agreeing and 35% and 33% agreeing, respectively (Fig. 4c and d). These results indicate that students’ perceptions on the effectiveness of the teaching and content were predominantly viewed as being excellent or good.

### Open Ended Survey Questions

To improve future sessions, we solicited narrative feedback in post-session surveys and the ECQ. We received 87 comments in total. Thematic analysis revealed the following frequencies: 4 regarding timing of sessions (early in the block or near the test), 6 regarding pre- and post-quizzes, 12 regarding practice questions and labeling exercises, 14 regarding the desire for longer sessions, 15 regarding learning tips and explanations provided by the facilitators, and 26 regarding the design of class materials. There were also 10 responses that were categorized as generally positive but without a clear content area.

Regarding the desire for longer sessions, students felt that sessions were rushed without adequate time to answer all the built-in practice questions and labeling exercises. Many said that it was challenging to have only one hour to review so much imaging and anatomy and that the end of sessions was sometimes interrupted by students coming into the classroom for the following class.

Students appreciated the practice questions within the sessions as a good way to address knowledge gaps. Challenges related to this theme centered around not having adequate time to review answers and not having the correct answers to pre- and post-session quizzes released. Class materials were also generally well received. Students preferred having images oriented from inferior to superior before reviewing content depicted on each image, having clinical concepts interwoven with imaging, and focusing on high-yield exam content.

## Discussion

Given the relative lack of imaging education in the preclinical years of medical school and the challenges associated with its implementation [1, 2], novel methods for addressing this gap are needed. A near-peer-led approach to supplementing imaging instruction is beneficial to both student learners and peer teachers and provides added benefits in that it doesn’t require extra space in the curriculum or time from radiologists. To meet a need in our curriculum, regular near-peer led imaging reviews were established and targeted M1s enrolled in FoCS, the first course medical students take upon matriculation at our institution. These review sessions were intended to supplement students’ anatomical and imaging knowledge. Results from this study showed that students not only performed better on post-session quizzes indicative of a short-term gain in knowledge, but highly valued these sessions for their content, delivery and perceived helpfulness for exam preparation.

Prior to the implementation of these sessions, imaging education at our institution consisted of interactive modules and one ninety-minute active learning class per block for a total of seven modules and seven active learning sessions. While students reported these to be helpful, they were limited by time with the most frequent student feedback concerning a desire for more time to review important anatomy and radiology topics [21]. Implementing new or expanding upon existing curricular elements is often a challenge due to availability of faculty [7]. The current study was designed to address the desire of students at our institution for more extensive and integrated anatomy and imaging education while fitting within the constraints that exist to implementing new curricular elements in radiological education [7, 8].

Near-peer teaching models have shown benefit to students at the clerkship [29, 30] and pre-clinical level [22]. Additionally, a review of near-peer teaching across many topics of study in medical school showed equal knowledge gain between near-peer and faculty-led sessions [31]. These data indicate that student learning is not hindered in a near-peer format and that it may even be beneficial. We found that students performed significantly better on the majority of post-session quizzes, and they felt more prepared to answer imaging and anatomy-related exam questions following the sessions. Prior work from our institution similarly found that first and second year medical students performed better on post-session quizzes following engagement in anatomy workshops designed to help prepare students for the USMLE Step1 exam [32]. In the current study, students performed significantly better on all post-session quizzes except for the upper limb block (Block 2). Here, performance on the post-session quiz, while higher than on the pre-session quiz, was not statistically significant. Indeed, there are several factors which may account for the lack of statistical significance, including the difficulty of the material and the timing of the near-peer- led review session. Regarding the difficulty, students had several muscles of the upper limb to identify on cross sections, likely making it more challenging to spatially orient on images. Regarding timing, the near-peer led session occurred one day after the faculty-led imaging session. While the timing was unavoidable due to other curricular constraints, it likely limited students’ abilities to effectively synthesize the material. When students answered a subset of these pre- and post-session quiz questions one month after the conclusion of FoCS, scores overall demonstrated knowledge retention of the tested concepts. This study is the first that we are aware of to investigate longer term knowledge retention following implementation of near-peer led review sessions. Students additionally reported that these sessions addressed knowledge gaps and reinforced preexisting knowledge and further reported better overall understanding of imaging and anatomy topics. These data, paired with consistently strong attendance at sessions, indicate a perceived benefit that these sessions had on student learning outcomes. It would be particularly relevant for future work to incorporate elements of spaced repetition of imaging and anatomy concepts to aid student understanding and long-term knowledge retention. A recent report by Yao et al. [33] offered guidance on how spaced repetition could potentially be incorporated into medical imaging education through use of Anki decks to aid efficiency in knowledge acquisition and retention.

If near-peer teaching is going to be effective in the long term, students must feel that they are engaging in sessions that will help them feel prepared for exams. Our review sessions led to students feeling more prepared for exam questions as evidenced from results in both the post-session surveys and the ECQ. Many students also reported using PowerPoint materials to study for exams. This demonstrates that students perceived the materials prepared by their near peers to be a valuable resource for exam preparation. This would support the notion that near-peer educators can be as effective as faculty to design and deliver high quality educational materials [30]. The majority of M1 students rated the near-peer instruction positively, with many leaving comments commending its structure and design. It’s likely that students viewed these sessions positively not only because of their educational value, but also because the M2 facilitators approached their instruction in a student-centered manner. Known as constructivist pedagogy [34], M2 facilitators built on M1 students’ prior anatomical and imaging knowledge by incorporating appropriate group-based learning activities to engage students in active learning. M2 facilitators further guided students on the application of their anatomical and imaging knowledge by connecting these with real-life clinical scenarios, another element of constructivist pedagogy [34]. Upon reflection, M2 instructors perceived that creating and leading these sessions expanded their knowledge of the material, M2 students also experienced strong satisfaction with session instruction. They perceived many tangible benefits of teaching including communication and presentation skill development, increased engagement with course material, and personal gratification of helping their peers learn the material.

Students may find near-peer teaching preferable to faculty-led teaching in some circumstances because they feel more comfortable with near-peers [30, 35]. In fact, one study reported that didactic sessions led by near-peers within a radiology clerkship were the highest rated sessions of the course [30]. When we asked whether learners were satisfied with sessions, the response was overwhelmingly positive towards the effectiveness of teaching, facilitator communication, session content and session delivery. These findings support the social congruence framework of this study, which posits that student learners feel more comfortable communicating with and thus, learning from near peers given the proximity in their training stage [24]. Specifically, within the context of social congruence, student learners have found that their near-peer teachers are more approachable and empathetic and thus supportive of their learning [24, 25], elements that were noted from the positive feedback associated with our study. The findings additionally support the cognitive congruence framework of this study in that near-peer teachers, having recently completed the imaging and anatomy components of the curriculum, were better able to explain these topics at the learners’ level. The cognitive congruence framework asserts that near peers are best suited to disseminate their knowledge as they can better identify with learners’ struggles and thus help them fill in their knowledge gaps [23, 25]. When given the opportunity to provide open-ended narrative feedback, students frequently commented positively on the built-in practice questions, pre- and post-session quizzes, learning tips from near peers within sessions and the desire for longer sessions. These findings further support the cognitive congruence framework in that near-peers were able to disseminate their knowledge in a manner that supported learners’ understanding and that near-peers were additionally able share learning tips and emphasize points that were important for exam preparation were valued by learners. With respect to imaging education, Oldan et al. [36] similarly demonstrated improved medical student satisfaction with imaging curricula delivered by radiology residents and Tung et al. [37] reported improved resident knowledge following resident-to-resident teaching in pediatric radiology, supporting the cognitive and social congruence frameworks of peer teaching.

There are key similarities and differences between this work and prior studies investigating near-peer teaching. In our study, second year medical students independently led review sessions as opposed to doing so alongside a resident or attending physician [22, 29]. Medema et al. [30] reported a similar model in which fourth year students, not directly accompanied by a resident or physician, led imaging review sessions for third year medical students on radiology clerkships. Despite the success of this design, many medical schools do not have a radiology clerkship for third year medical students so integrating near-peer radiology teaching should ideally happen prior to the third year, as was done in our study. In several studies, including ours, near peers facilitate review sessions, adding to the established framework of the existing medical curriculum [22, 29, 30]. Ramakrishnan et al. [29] described an internal medicine pre-clerkship bootcamp led by a post-clerkship medical student and resident. This 1-hour session was intended to help students transition from the classroom to clerkships and since it was led by near-peers, it had the advantage of offering support and guidance from facilitators who recently completed the clerkship. The group not only covered topics such as efficient chart review and patient presentation but also created a survival guide meant to serve as a reference for students during the clerkship. Similar to Ramakrishnan [29], we gave students access to session materials for use as a study guide and reference.

The implementation of these sessions at our institution carried many benefits as well as a few challenges. First, the use of near-peer educators allowed these sessions to occur outside of designated curricular hours, when time may be more limited for faculty. Another is that second year medical students are better able to disseminate information about where and when these sessions will be held given interpersonal connections with other students. Additionally, these sessions provide an opportunity for spaced repetition in both imaging and anatomy. One challenge of implementation was the relatively large amount of work to get these sessions started. Creating the presentation materials takes time and effort which can be challenging for students to find during a busy curriculum. This is especially true with second year medical students who are preparing to take the USMLE Step 1 exam. This effort, however, is mostly confined to the first year in which these sessions are run since the slides can be used in future years and edited as needed.

As a future goal, the impact of near-peer led imaging review sessions on students’ long-term imaging knowledge could be assessed at multiple time points (e.g. at the end of FoCS, end of the first year and end of the second year or post USMLE Step 1). These additional time frames for assessment would provide a richer data set from which to investigate how imaging knowledge retention not only changes over time, but whether attendance in near-peer-led imaging sessions leads to better knowledge retention in comparison to peers who did not attend. This assessment would focus on imaging knowledge from radiographs, CTs, MRIs and angiograms as opposed to anatomical knowledge assessed in our ECQ. Another interesting future goal would be to conduct focus group sessions with student attendees. These focus groups would be timed so that they occur toward the end of clerkships, giving students the opportunity to implement and practice their medical imaging knowledge. The focus groups would be designed to determine students’ perceptions on the impact of near-peer led-imaging reviews on their perceived imaging knowledge, confidence in their knowledge and accuracy of diagnosis based on imaging findings upon entering their clerkships.

In conclusion, the results demonstrate a practical approach in which near-peer teaching can be incorporated into an existing medical imaging curriculum. Students performed better on post-tests associated with each near-peer-led review session and perceived that their anatomical and imaging knowledge along with exam preparedness improved following attendance in near-peer-led review sessions. While student feedback was positive overall, many student respondents commented on the desire for longer review sessions. To address this point, a future goal will aim to remove the post-session survey from each session and give one survey at the end of the course. Another way to lengthen the sessions will be to schedule later in the day after students finish their afternoon classes. Finally, we hope to continue these sessions and thus plan to recruit interested M2 students each year to fill the role of session leader. We will continue data collection and analysis in the coming years to not only ensure that these sessions are useful for M1s, but to also assess whether these sessions impact student interest in pursuing radiology (i.e. student enrolment in radiology professional development weeks, rotations and specialty matching). The ability to implement a near-peer-led imaging program with strong positive outcomes offers a solution to the challenges associated with curricular and faculty-time constraints and benefits all parties involved including learners, peers, faculty and administrators.

### Limitations

While this study provided important insights into the benefits of a near-peer led approach toward anatomical and imaging education, there are some important limitations. Of importance is the absence of a control group against which to test a primary outcome of learning benefit with and without these sessions. As such, concrete conclusions about the direct role of these sessions in student academic performance cannot be made. Differences in student interest, knowledge or background were not accounted for within this study. This could potentially mean that many of the students who attended these sessions had an interest in radiology or were more intrinsically motivated to ensure better exam performance. Along these lines, an average of only ~ 21% of student participants completed post-session surveys. It’s possible that these students were most interested and/or motivated by the topics and thus were more likely to engage in post-session surveys. The present study involved the use of the same pre- and post-tests. While this was done to reliably assess knowledge before and after the intervention, students may have done better on the post-test as they clued in on certain pre-test details during the session. Furthermore, it may be difficult to generalize the effects of the intervention for studies in which no pre-test is given. Another important consideration with the pre/post-test quizzes is that not all students who took the pre-test also took the post-test. This can reduce statistical power and may also have biased results. Furthermore, ordinal data were assumed to be continuous for statistical analysis, which may introduce bias from variability in Likert scale interpretation by students. An additional limitation of the study is the timing in which the ECQ, intended to capture longer-term anatomy knowledge retention, was administered. Since students took the ECQ shortly after their winter break, there was a one-month period of time that elapsed between completing their coursework and taking the ECQ. To best capture knowledge retention over time, this would have been best done over multiple time periods and as such, is of interest for future studies. Despite these limitations, the data presented here support the idea that near-peer teaching is a useful approach toward preclinical anatomical imaging education.

## Supplementary Information

Below is the link to the electronic supplementary material.

Supplementary file1 (PPTX 57 KB)

Supplementary file2 (DOCX 2287 KB)

Supplementary file3 (DOCX 25 KB)

Supplementary file4 (DOCX 20 KB)

## Data Availability

The authors declare that they had full access to all data in this study and the authors take complete responsibility for the integrity of the data and the accuracy of the data analysis.

## References

[1] Webb EM, Naeger DM, McNulty NJ, Straus CM. Needs assessment for standardized medical student imaging education: review of the literature and a survey of deans and chairs. Acad Radiol. 2015;22(10):1214–20. 26259548 10.1016/j.acra.2015.03.020

[2] Schiller PT, Phillips AW, Straus CM. Radiology education in medical school and residency: the views and needs of program directors. Acad Radiol. 2018;25(10):1333–43. 29748045 10.1016/j.acra.2018.04.004

[3] Chew C, O’Dwyer PJ, Sandilands E. Radiology for medical students: do we teach enough? A national study. Br J Radiol. 2021;94(1119):20201308. 33560874 10.1259/bjr.20201308 PMC8011254

[4] Saha A, Roland RA, Hartman MS, Daffner RH. Radiology medical student education: an outcome-based survey of PGY-1 residents. Acad Radiol. 2013;20(3):284–9. 23452472 10.1016/j.acra.2012.10.006

[5] Prezzia C, Vorona G, Greenspan R. Fourth-year medical student opinions and basic knowledge regarding the field of radiology. Acad Radiol. 2013;20(3):272–83. 23452471 10.1016/j.acra.2012.10.004

[6] Rohren SA, Kamel S, Khan ZA, Patel P, Ghannam S, Gopal A, et al. A call to action; national survey of teaching radiology curriculum to medical students. J Clin Imaging Sci. 2022;12:57. 36325497 10.25259/JCIS_36_2022 PMC9610045

[7] Straus CM, Webb EM, Kondo KL, Phillips AW, Naeger DM, Carrico CW, et al. Medical student radiology education: summary and recommendations from a national survey of medical school and radiology department leadership. J Am Coll Radiol. 2014;11(6):606–10. 24713496 10.1016/j.jacr.2014.01.012

[8] Branstetter BF, Faix LE, Humphrey AL, Schumann JB. Preclinical medical student training in radiology: the effect of early exposure. AJR Am J Roentgenol. 2007;188(1):W9-14. 17179333 10.2214/AJR.05.2139

[9] Lufler RS, Zumwalt AC, Romney CA, Hoagland TM. Incorporating radiology into medical gross anatomy: does the use of cadaver CT scans improve students’ academic performance in anatomy. Anat Sci Educ. 2010;3(2):56–63. 20213692 10.1002/ase.141

[10] Erkonen WE, Albanese MA, Smith WL, Pantazis NJ. Effectiveness of teaching radiologic image interpretation in gross anatomy. A long-term follow-up. Invest Radiol. 1992;27(3):264–6. 1551779 10.1097/00004424-199203000-00016

[11] Erkonen WE, Albanese MA, Smith WL, Pantazis NJ. Gross anatomy instruction with diagnostic images. Invest Radiol. 1990;25(3):292–4. 2332316 10.1097/00004424-199003000-00018

[12] de Barros N, Rodrigues CJ, Rodrigues AJ, de Negri Germano MA, Cerri GG. The value of teaching sectional anatomy to improve CT scan interpretation. Clin Anat. 2001;14(1):36–41. 11135396 10.1002/1098-2353(200101)14:1<36::AID-CA1006>3.0.CO;2-G

[13] Brown B, Adhikari S, Marx J, Lander L, Todd GL. Introduction of ultrasound into gross anatomy curriculum: perceptions of medical students. J Emerg Med. 2012;43(6):1098–102. 22459597 10.1016/j.jemermed.2012.01.041

[14] Dreher SM, DePhilip R, Bahner D. Ultrasound exposure during gross anatomy. J Emerg Med. 2014;46(2):231–40. 24113480 10.1016/j.jemermed.2013.08.028

[15] Grignon B, Oldrini G, Walter F. Teaching medical anatomy: what is the role of imaging today. Surg Radiol Anat. 2016;38(2):253–60. 26298830 10.1007/s00276-015-1548-y

[16] Khalil MK, Payer AF, Johnson TE. Effectiveness of using cross-sections in the recognition of anatomical structures in radiological images. Anat Rec B New Anat. 2005;283(1):9–13. 15761832 10.1002/ar.b.20053

[17] Yang C, Davis K, Head M, Huck NA, Irani T, Ovakimyan A, et al. Collaborative learning communities with medical students as teachers. J Med Educ Curric Dev. 2023;10:23821205231183880. 37362582 10.1177/23821205231183878 PMC10286217

[18] Burgess A, McGregor D, Mellis C. Medical students as peer tutors: a systematic review. BMC Med Educ. 2014;9(14):115. 10.1186/1472-6920-14-115 PMC4237985 24912500

[19] Shenoy A, Petersen KH. Peer tutoring in preclinical medical education: a review of the literature. Med Sci Educ. 2020;30(1):537–44. 34457698 10.1007/s40670-019-00895-y PMC8368558

[20] Burgess A, Dornan T, Clarke AJ, Menezes A, Mellis C. Peer tutoring in a medical school: perceptions of tutors and tutees. BMC Med Educ. 2016;8(16):85. 10.1186/s12909-016-0589-1 PMC4784332 26956642

[21] Afshari S, Lythgoe J, Zhou M, Barton C, Warfield A, Walsh R, et al. An interdisciplinary approach toward developing an engaging and clinically relevant medical imaging curriculum. Acad Radiol. 2025;32(2):1127–37. 39505588 10.1016/j.acra.2024.10.005

[22] Naeger DM, Conrad M, Nguyen J, Kohi MP, Webb EM. Students teaching students: evaluation of a “near-peer” teaching experience. Acad Radiol. 2013;20(9):1177–82. 23810649 10.1016/j.acra.2013.04.004

[23] Lockspeiser TM, O’Sullivan P, Teherani A, et al. Understanding the experience of being taught by peers: the value of social and cognitive congruence. Advances Health Sci Educ. 2008;13(3):361–72. 10.1007/s10459-006-9049-8 17124627

[24] Schmidt HG, Moust JH. What makes a tutor effective? A structural-equations modeling approach to learning in problem-based curricula. Acad Med. 1995;70(8):708–14. 7646747 10.1097/00001888-199508000-00015

[25] Ten Cate O, Durning S. Peer teaching in medical education: twelve reasons to move from theory to practice. Med Teach. 2007;29(6):591–9. 17922354 10.1080/01421590701606799

[26] Loda T, Erschens R, Loenneker H, Keifenheim KE, Nikendei C, Junne F, et al. Cognitive and social congruence in peer-assisted learning - A scoping review. PLoS One. 2019;14(9):0222224. 10.1371/journal.pone.0222224. PMC6733464 31498826

[27] Brown W, Afshari S, Zhou M, Lythgoe J, Walsh R, Hielscher AC. Living and post-mortem CT scans in the gross anatomy lab: a study investigating differences in first-year medical students’ exam performance and perceptions. Anat Sci Educ. 2024;17(3):468–82. 38213130 10.1002/ase.2371

[28] Braun V, Clark V. Toward good practice in thematic analysis: avoiding common problems and be(com)ing a *knowing* researcher. Int J Transgend Health. 2022;24(1):1–6. 10.1080/26895269.2022.2129597. 36713144 PMC9879167

[29] Ramakrishnan D, Ilagan-Ying YC, Bollinger B, Dunne D. Teaching sideways: implementing a peer-led medical student orientation bootcamp and survival guide for internal medicine clerkship. South Med J. 2024;117(1):1–6. 38151243 10.14423/SMJ.0000000000001638

[30] Medema AM, Goins SM, French RJ, Martin JG. Near-peer paradigms in medical school: integrating student teaching assistants into a core radiology clerkship. Acad Radiol. 2024;31(8):3464–70. 38862348 10.1016/j.acra.2024.04.050

[31] Benè KL, Bergus G. When learners become teachers: a review of peer teaching in medical student education. Fam Med. 2014;46(10):783–7. 25646829

[32] Ricci A, Minearo I, Hielscher A. Focused anatomy workshops for clerkships and the USMLE Step 1 examination. Anat Sci Educ. 2025;18(1):77–86. 39578709 10.1002/ase.2536 PMC11669074

[33] Yao K, Nguyen J, Mathur M. Spaced repetition learning in radiology education: exploring its potential and practical application. J Am Coll Radiol. 2024;22:15–21. 39612969 10.1016/j.jacr.2024.11.020

[34] Do HN, Do BN, Nguyen MH. How do constructivism learning environments generate better motivation and learning strategies? The design science approach. Heliyon. 2023;9(12):e22862. 10.1016/j.heliyon.2023.e22862. 38125439 PMC10730747

[35] Khapre M, Deol R, Sharma A, Badyal D. Near-peer tutor: a solution for quality medical education in faculty constraint setting. Cureus. 2021;13(7):e16416. 34422460 10.7759/cureus.16416 PMC8369978

[36] Oldan JD, Jordan SG, Wallace J, Campbell J, Fordham LA, Beck Dallaghan GL. Near-peer teaching in radiology symposia: a success story in residents as teachers. J Med Educ Curric Dev. 2023. 10.1177/23821205231162459. PMC9996712 36911752

[37] Tung EL, Shailam R, Tung MG, Barton K. A virtual multi-institution pediatric radiology peer teaching conference improves knowledge of educators. Curr Probl Diagn Radiol. 2025;54(3):377–81. 39098404 10.1067/j.cpradiol.2024.07.017

