# Supplementary material for: Near-Peer Teaching to Integrate Anatomical Imaging into the Medical School Curriculum
Source: Med Sci Educ. 2026 Jun 3;36(4):1911–22. doi: 10.1007/s40670-026-02760-1 (PMC13582774; doi:10.1007/s40670-026-02760-1)
Supplement: Supplementary file 2 [file 40670_2026_2760_MOESM3_ESM.docx]

**Supplementary Figure 3**

**a.**

**Block 1 Pre/Post Quiz**

1) Where does the C8 nerve exit in relation to the C7 Vertebral body?
   a. Intervertebral foramen between C7 and C8
   b. Intervertebral foramen between C8 and T1
   c. Between C7 and C8 vertebral bodies
   d. Between C8 and T1 vertebral bodies
2) What are the two parts of the intervertebral disc?
   a. Nucleus pulposus and annulus fibrosus
   b. Paravertebral and prevertebral
   c. Peripheral disc and medial disc
   d. Anterior and posterior longitudinal ligaments
3) Which direction is most common for a disc herniation
   a. Anterior
   b. Posterior
   c. Lateral
   d. They are all equally likely
4) Which artery is most at risk of ligation following a transverse fracture to the surgical neck of the humerus?
   a. Radial artery
   b. Subclavian artery
   c. Deep brachial artery
   d. Circumflex scapular artery
   e. Posterior humeral circumflex
5) What muscles make up the rotator cuff (4)
   a. Teres Minor
   b. Subscapularis
   c. Supraspinatus
   d. Infraspinatus
6) A patient walks into your clinic with a concerned look on his face saying that he feels like he hurt himself while lifting weights recently. On physical exam he is unable to initiate abduction of his shoulder. Which muscle is most likely injured in this patient?
   a. Deltoid
   b. Trapezius
   c. Supraspinatus
   d. Teres major
   e. Latissimus dorsi
7) At what spinal level is the conus medullaris most often found in an adult?
   a. L 3/4
   b. L 3/4
   c. S 1/2
   d. L 1/2
8) What is the last ligamentous layer pierced by a needle during a spinal tap (sampling CSF from spinal cord? Bonus: in what space is CSF found?
   a. Interspinous ligament
   b. Annulus fibrosus
   c. Ligamentum flavum (Bonus: CSF is found within the subarachnoid space)
   d. Supraspinous ligament
9) Which of the following bony structures is found on the scapula?
   a. Manubrium
   b. Infraglenoid tubercle
   c. Radial fossa
   d. Anatomical neck

**Block 2 Pre/Post Quiz**

1) Fracture to the medial epicondyle of the humerus is associated with damage to what other structure?
   a. Radial nerve
   b. Median nerve
   c. Ulnar nerve
   d. Anterior interosseus nerve
   e. Axillary nerve
2) Fracture to what part of the humerus is most likely to result in radial nerve damage? What other structure is also at most risk of damage?
   a. Midshaft humerus and deep brachial artery
   b. Surgical neck and ulnar artery
   c. Midshaft humerus and radial artery
   d. Lateral epicondyle and brachial artery
   e. Surgical neck and posterior humoral circumflex artery
3) A patient who loses the ability to abduct and adduct their fingers likely has damaged a nerve in what anatomical area?
   a. Carpal tunnel
   b. Thenar eminence
   c. Lateral epicondyle
   d. Guyon’s canal
4) In general the flexors and extensors of the forearm attach to what bony structures respectively?
   a. Flexors attach to the ulna, extensors to the radius
   b. Flexors attach to the medial epicondyle, extensors to the lateral epicondyle
   c. Flexors attach to the lateral epicondyle, extensors to the coronoid process
   d. They all attach to different spots with no discernable pattern
5) If a patient were to trip and fall landing on an outstretched hand which of the carpal bones would be most likely damaged? Bonus: what space can you find this bone in?
   a. Lunate
   b. Hamate
   c. Triquetrum
   d. Pisiform
   e. Scaphoid (anatomic Snuff Box)
6) What nerve injury is associated with a winged scapula, what spinal roots feed this nerve?
   a. Long thoracic nerve, C5-C7
   b. Thoracodorsal nerve, C6-C8
   c. Dorsal Scapular nerve, C5
   d. Superior and inferior subscapular nerves, both C5-C6
7) A patient presenting with an adducted, extended and medially rotated arm likely has damage to which spinal nerves?
   a. C8-T1
   b. C5-C6
   c. C7-T1
   d. C6-C8
8) Anterior dislocation of the humerus at the glenohumeral joint would risk damage to which nerve?
   a. Radial nerve
   b. Axillary nerve
   c. Median nerve
   d. Musculocutaneous nerve

**Block 3 Pre/Post Quiz:**

1) What external landmark can be used to find the level of the aortic arch?
   a. Xiphoid process
   b. Sternal angle of louis
   c. Jugular notch
   d. Nipple line
   e. Sternoclavicular joint
2) The posterior intercostal arteries come off of what larger vascular structure?
   a. Internal thoracic artery
   b. Musculophrenic artery
   c. Descending aorta
   d. Subclavian arteries bilaterally
3) Where can the anterior interosseus vessels and nerve be found in relation to the related rib and in what orientation from superior to inferior are they? N=nerve, A=artery, V=vein
   a. Costal groove at the top of the rib in the order of NAV
   b. Along the middle part of the inner surface of the rib in the order of NAV
   c. In the costal groove along the bottom of the rib in the orientation of VAN
   d. Along the middle part of the inner surface of the rib in the order of VAN
4) What is the orientation of the phrenic nerve and vagus nerve in relation to the roots of the lungs?
   a. Phrenic nerve runs anterior, and vagus nerve runs posterior
   b. Both run posterior
   c. Both run anterior
   d. Phrenic nerve runs posterior, and vagus nerve runs anterior
5) What is the orientation of the pulmonary artery to the bronchus on each side? RPA= right pulmonary artery, LPA= left pulmonary artery, RMA = right main bronchus, LMA= left main bronchus
   a. RPA posterior to RMB, LPA anterior to LMB
   b. RPA inferior to RMB, LPA inferior to LMB
   c. RPA superior to RMB, LPA anterior to LMB
   d. RPA anterior to RMB, LPA superior to LMB
6) Which of the thoracic cage muscles aids in inspiration?
   a. Internal intercostal
   b. External intercostals
   c. Innermost intercostals
   d. Transversus thoracis
7) A patient aspirates laying in the supine position, where is that foreign material most likely to be found?
   a. Right Lower Lobe
   b. Left Lower Lobe
   c. Left lower lobe
   d. Right Middle lobe
   e. Right upper lobe
8) Which coronary artery supplies the pace making cells of the heart in the majority of people?
   a. Left anterior descending artery
   b. Left circumflex artery
   c. Right coronary artery
   d. Left coronary artery

**Block 4 Pre/Post Quiz:**

1) How can the pain from appendicitis best be described
   a. Pain is immediately localized over McBurney’s point
   b. Pain begins in the LLQ then moves into the RLQ 1/3 of the ASIS-umbilicus distance
   c. Pain is originally periumbilical then is localized in the RLQ at McBurney’s point
   d. Cannot be localized because visceral pain is poorly localizable
2) What artery is most directly at risk of bleeding in a patient with duodenal ulcer?
   a. Common hepatic artery
   b. Superior mesenteric artery
   c. Pancreaticoduodenal artery
   d. Gastroduodenal artery
   e. Splenic artery
   f. Inferior mesenteric artery
3) What dermatome supplies the umbilicus?
   a. T6
   b. T4
   c. T12
   d. T10
4) A gallstone located where would lead to pancreatic outflow obstruction?
   a. Common bile duct
   b. Ampulla of Vater
   c. Cystic duct
   d. All of the above
5) The rectum above the pectinate line is drained by what vein as a part of which venous system?
   a. Inferior rectal vein, portal venous system
   b. Inferior rectal vein, systemic venous system
   c. Superior rectal vein, portal venous system
   d. Superior rectal vein, systemic venous system
6) Which veins come together to form the portal vein?
   a. Splenic vein and SMV
   b. Renal vein and IMV
   c. IMV and SMV
   d. Renal vein and splenic vein
7) What are the main arterial branches of the Celiac trunk?
   a. Splenic, left gastric, SMA
   b. Common hepatic, splenic, left gastric
   c. Common hepatic, SMA, IMA
   d. Common hepatic, splenic, left renal
8) What section of the duodenum do pancreatic and bilious juices drain into?
   a. 1^st^
   b. 2^nd^
   c. 3^rd^
   d. 4^th^
   e. 5^th^

**Block 5 Pre/Post Quiz:**

1) Which nerves are responsible for the pupillary light reflex?
   a. Oculomotor afferent and optic efferent
   b. Opthalmic afferent and oculomotor efferent
   c. Optic Afferent and oculomotor efferent
   d. Optic afferent and ophthalmic efferent
2) Which type in intracranial bleed is associated with tearing of bridging veins?
   a. Epidural
   b. Subdural
   c. Subarachnoid
   d. Intraparenchymal
3) Down, out, and dilated pupil along with ptosis of the eyelid indicates damage to what cranial nerve?
   a. Optic
   b. Abducens
   c. Trochlear
   d. Ophthalmic
   e. Oculomotor
4) The nerve innervating the muscles of mastication travels through what opening in the skull?
   a. Foramen rotundum
   b. Foramen spinosum
   c. Superior orbital fissure
   d. Foramen Ovale
5) Which of the following cranial nerves is associated with damage following carotid endarterectomy?
   a. CNIX
   b. CNVIII
   c. CNVII
   d. CNVI
6) How do the vertebral arteries enter the skull?
   a. Through the vertebral canal
   b. Through the hypoglossal canal
   c. Through Foramen Magnum
   d. Through Jugular foramen
7) What separates the cerebellum from the cerebrum?
   a. Tentorium Cerebelli
   b. Falx Cerebri
   c. Corpus callosum
   d. Only CSF
8) Which of the following is a correct order of flow through the dural venous sinuses?
   a. Superior sagittal 🡪 confluence 🡪 transverse 🡪 sigmoid
   b. Sigmoid 🡪 transverse 🡪 confluence 🡪 superior sagittal
   c. Transverse 🡪 sigmoid 🡪 superior sagittal 🡪confluence
   d. Confluence 🡪 transverse 🡪 Superior sagittal 🡪 sigmoid

**Block 6**

1) What is the relation of the uterine artery to the ureter in the female pelvis?
   a. Ureter runs alongside the uterine artery
   b. Ureter runs under the uterine artery
   c. Uterine artery runs under the ureter
   d. The do not intersect
2) What are the spinal nerve levels of the pelvic splanchnic nerves?
   a. L2-L4
   b. L3-L5
   c. S1-S3
   d. S2-S4
3) What are the spinal nerve roots of the sciatic nerve?
   a. L2-S3
   b. L4-S3
   c. S1-S4
   d. L2-L4
4) What nerve runs through both the greater and lesser sciatic foramina?
   a. Pudendal nerve
   b. Obturator nerve
   c. Sciatic nerve
   d. Superior gluteal nerve
   e. Inferior gluteal nerve
5) Which ligament in the female pelvis runs through the inguinal canal to connect to the labia majora?
   a. Broad ligament
   b. Suspensory ligament
   c. Ovarian ligament
   d. Round ligament
6) From uterus to ovary what is the order of the parts of the uterine tube
   a. Isthmus 🡪ampulla 🡪 fimbriae 🡪 infundibulum
   b. Ampulla 🡪 infundibulum 🡪 isthmus 🡪 fimbriae
   c. Isthmus 🡪ampulla 🡪infundibulum 🡪 fimbriae
   d. Infundibulum 🡪 ampulla 🡪 Isthmus 🡪 fimbriae
7) What is the last layer that a spinal block needle pierces?
   a. Dura mater
   b. Arachnoid mater
   c. Ligamentum flavum
   d. Pia mater
8) Congenital hydrocele is due to patency of what structure
   a. Processus vaginalis
   b. Tunica albuginea
   c. Ischiocavernosus
   d. Corpus spongiosum

**Block 7**

1) The obturator nerve supplies which muscle group of the thigh?
   a. Abductors
   b. Flexors
   c. Adductors
   d. Extensors
2) From lateral to medial what the correct orientation of the femoral neurovascular structures?
   a. Lymph, vein, artery, nerve
   b. Nerve, artery, vein, lymph
   c. Artery, vein, nerve, lymph
   d. Nerve, vein, artery, lymph
3) What nerve supplies the gluteus maximus muscle?
   e. Superior gluteal nerve
   f. Inferior gluteal nerve
   g. Pudendal nerve
   h. Obturator nerve
   i. Sciatic nerve
4) What ligament of the knee prevents anterior translation of the tibia on the femur?
   a. ACL
   b. MCL
   c. PCL
   d. LCL
5) The origin of the sciatic nerve is found immediately deep to what muscle?
   a. Gluteus minimus
   b. Piriformis
   c. Gluteus Medius
   d. Obturator internus
6) What nerve innervates the structures of the anterior compartment of the lower leg?
   a. Common fibular nerve
   b. Tibial nerve
   c. Deep fibular nerve
   d. Superficial fibular nerve
   e. Femoral nerve
7) What ankle ligament is the most common to be sprained via an inversion injury?
   a. Deltoid ligament
   b. Anterior talofibular ligament
   c. Posterior talofibular ligament
   d. Anterior inferior tibiofibular ligament
8) Sensory loss in what dermatome will lead to loss of the patellar reflex?
   a. L5
   b. S1
   c. S2
   d. L4

**b.**

**Post-Session Survey (same for all blocks):**

**Optional Imaging Session Survey:**

1. How would you rate the effectiveness of the teaching in this session?

1: Poor

2: Needs improvement

3: Adequate

4: Good

5: Excellent

2. How would you rate your preparedness to answer the quiz questions before the session?

1: Not ready at all

2: Getting there

3: Neutral

4: Mostly ready

5: Completely ready

3. How would you rate your preparedness to answer the quiz questions after the session?

1: Not ready at all

2: Getting there

3: Neutral

4: Mostly ready

5: Completely ready

4. Following the session, how ready do you feel to answer anatomy and imaging based questions on the upcoming test?

1: Not ready at all

2: Getting there

3: Neutral

4: Mostly ready

5: Completely ready

5. Did this session help to clarify what material is most important to your learning for this block?

1: Yes

2: No

3: Not sure

6. What do you feel worked well in the session and what did not work well?
7. Do you have any other comments, thoughts or suggestions?
