# Supplementary material for: Near-Peer Teaching to Integrate Anatomical Imaging into the Medical School Curriculum
Source: Med Sci Educ. 2026 Jun 3;36(4):1911–22. doi: 10.1007/s40670-026-02760-1 (PMC13582774; doi:10.1007/s40670-026-02760-1)
Supplement: Supplementary file 3 [file 40670_2026_2760_MOESM4_ESM.docx]

**Supplementary Figure 4**

Semester End Quiz:

1) Where does the C8 nerve exit in relation to the C7 Vertebral body?
   a. Intervertebral foramen between C7 and C8
   b. Intervertebral foramen between C7 and T1
   c. Between C7 and C8 vertebral bodies
   d. Between C8 and T1 vertebral bodies
2) Which artery is most at risk of ligation following a transverse fracture to the surgical neck of the humerus?
   a. Radial artery
   b. Subclavian artery
   c. Deep brachial artery
   d. Circumflex scapular artery
   e. Posterior humeral circumflex
1) Fracture to what part of the humerus is most likely to result in radial nerve damage? What other structure is also at most risk of damage?
   a. Midshaft humerus and deep brachial artery
   b. Surgical neck and ulnar artery
   c. Midshaft humerus and radial artery
   d. Lateral epicondyle and brachial artery
   e. Surgical neck and posterior humoral circumflex artery
2) Anterior dislocation of the humerus at the glenohumeral joint would risk damage to which nerve?
   a. Radial nerve
   b. Axillary nerve
   c. Median nerve
   d. Musculocutaneous nerve
1) What is the orientation of the phrenic nerve and vagus nerve in relation to the roots of the lungs?
   a. Phrenic nerve runs anterior, and vagus nerve runs posterior
   b. Both run posterior
   c. Both run anterior
   d. Phrenic nerve runs posterior, and vagus nerve runs anterior
2) Which coronary artery supplies the pace making cells of the heart in the majority of people?
   a. Left anterior descending artery
   b. Left circumflex artery
   c. Right coronary artery
   d. Left coronary artery
1) What artery is most directly at risk of bleeding in a patient with duodenal ulcer?
   a. Common hepatic artery
   b. Superior mesenteric artery
   c. Pancreaticoduodenal artery
   d. Gastroduodenal artery
   e. Splenic artery
   f. Inferior mesenteric artery
2) What are the main arterial branches of the Celiac trunk?
   a. Splenic, left gastric, SMA
   b. Common hepatic, splenic, left gastric
   c. Common hepatic, SMA, IMA
   d. Common hepatic, splenic, left renal
1) Which nerves are responsible for the pupillary light reflex?
   a. Oculomotor afferent and optic efferent
   b. Ophthalmic afferent and oculomotor efferent
   c. Optic afferent and oculomotor efferent
   d. Optic afferent and ophthalmic efferent
2) Which type of intracranial bleed is associated with tearing of bridging veins?
   a. Epidural
   b. Subdural
   c. Subarachnoid
   d. Intraparenchymal
1) Congenital testicular hydrocele is due to patency of what structure?
   a. Processus vaginalis
   b. Tunica albuginea
   c. Ischiocavernosus
   d. Corpus spongiosum
2) What is the relation of the uterine artery to the ureter in the female pelvis?
   a. Ureter runs alongside the uterine artery
   b. Ureter runs under the uterine artery
   c. Uterine artery runs under the ureter
   d. The do not intersect
1) The origin of the sciatic nerve is found immediately deep to what muscle?
   a. Gluteus minimus
   b. Piriformis
   c. Gluteus Medius
   d. Obturator internus
2) What nerve innervates the structures of the anterior compartment of the lower leg?
   a. Common fibular nerve
   b. Tibial nerve
   c. Deep fibular nerve
   d. Superficial fibular nerve
   e. Femoral nerve

Question 1: Which of the following imaging peer-led review sessions did you attend?

Block 1 Back: Yes/No

Block 2 Upper limb: Yes/No

Block 3 Thorax: Yes/No

Block 4 Abdomen: Yes/No

Block 5 Head/Neck: Yes/No

Block 6 Pelvis: Yes/No

Block 7 Lower limb: Yes/No

Question 2: Please indicate your agreement with the following statement: The imaging review sessions were effective in reinforcing my anatomical knowledge.

a. Strongly disagree
b. Disagree
c. Neither agree nor disagree
d. Agree
e. Strongly agree

Question 3: Please indicate your agreement with the following statement: The imaging review sessions were effective in helping me address knowledge gaps.

a. Strongly disagree
b. Disagree
c. Neither agree nor disagree
d. Agree
e. Strongly agree

Question 4: Please indicate your agreement with the following statement: The content of each session was presented in a logical fashion that enabled me to build upon my knowledge.

a. Strongly disagree
b. Disagree
c. Neither agree nor disagree
d. Agree
e. Strongly agree

Question 5: Please indicate your agreement with the following statement: The peer facilitators effectively communicated the session content.

a. Strongly disagree
b. Disagree
c. Neither agree nor disagree
d. Agree
e. Strongly agree

Question 6: Please Indicate your agreement with the following statement: I am satisfied with the content of the imaging review sessions.

a. Strongly disagree
b. Disagree
c. Neither agree nor disagree
d. Agree
e. Strongly agree

Question 7: Please Indicate your agreement with the following statement: I am satisfied with the delivery of the imaging review sessions.

a. Strongly disagree
b. Disagree
c. Neither agree nor disagree
d. Agree
e. Strongly agree

Question 8: Please indicate your agreement with the following statement: The imaging review sessions prepared me for taking the secure exam.

a. Strongly disagree
b. Disagree
c. Neither agree nor disagree
d. Agree
e. Strongly agree

Question 9: Please indicate your agreement with the following statement: The imaging review sessions prepared me for taking the anatomy practical.

a. Strongly disagree
b. Disagree
c. Neither agree nor disagree
d. Agree
e. Strongly agree

Question 10: Please indicate your agreement with the following statement: My understanding of anatomy and imaging was very strong before attending one or more review sessions?

a. Strongly disagree
b. Disagree
c. Neither agree nor disagree
d. Agree
e. Strongly agree

Question 11: Please indicate your agreement with the following statement: My understanding of anatomy and imaging was very strong after attending one or more review sessions?

a. Strongly disagree
b. Disagree
c. Neither agree nor disagree
d. Agree
e. Strongly agree

Question 12: How often did you use the imaging review PowerPoints to study for your exams?

1. Never
2. Rarely
3. Sometimes
4. Often
5. Always

Question 13: Is there anything you would suggest to make imaging review sessions more successful for future students?

Please use this space to answer the question
