# Supplementary material for: Near-Peer Teaching to Integrate Anatomical Imaging into the Medical School Curriculum
Source: Med Sci Educ. 2026 Jun 3;36(4):1911–22. doi: 10.1007/s40670-026-02760-1 (PMC13582774; doi:10.1007/s40670-026-02760-1)
Supplement: Supplementary file 4 [file 40670_2026_2760_MOESM2_ESM.docx]

**Supplementary Figure 2**
